# A gap-free, telomere-to-telomere chromosome-scale genome assembly of the mangrove red snapper, *Lutjanus argentimaculatus*

**DOI:** 10.1038/s41597-026-08016-2

**Published:** 2026-08-05

**Authors:** Yang Xiang, Zhencheng Lu, Haoling Jiang, Yusong Guo, Zhongduo Wang

**Affiliations:** 1 Key Laboratory of Aquaculture in South China Sea for Aquatic Economic Animal of Guangdong Higher Education Institutes, Fisheries College, Guangdong Ocean University, Zhanjiang, China; 2 Guangdong Provincial Key Laboratory of Aquatic Animal Disease Control and Healthy Culture, Fisheries College, Guangdong Ocean University, Zhanjiang, China

## Abstract

The mangrove red snapper (*Lutjanus argentimaculatus*) is a commercially important marine fish species in the Indo-Pacific region. Despite its significant economic value for aquaculture, existing genomic resources remain fragmented, limiting the advancement of molecular breeding and functional genomic studies. Here, we present a gap-free, telomere-to-telomere (T2T) genome assembly of *L. argentimaculatus*, generated using a hybrid approach combining PacBio HiFi, Oxford Nanopore ultra-long reads and Hi-C technology. The resulting assembly comprises exactly 24 scaffolds spanning 1.03 Gb, perfectly matching the haploid chromosome number with a contig N50 of 46.17 Mb. Notably, this assembly resolves all physical gaps present in previous versions, achieving a BUSCO completeness score of 98.2%. Comprehensive genome annotation successfully predicted 23,167 protein-coding genes. Among these, 22,067 genes (95.25%) were functionally annotated across major public databases, including eggNOG, InterPro, and Swiss-Prot. Furthermore, structural analysis successfully identified 19 telomeres and 20 centromeres, validating the chromosomal integrity. This high-fidelity, gap-free reference genome provides a robust foundation for comparative genomics, population genetics, and the genetic improvement of *Lutjanidae* species.

## Background & Summary

The mangrove red snapper (*Lutjanus argentimaculatus*) is a marine ray-finned fish belonging to family *Lutjanidae*^1^. It is widely distributed across the mangrove estuaries of the Indo-Pacific region, including Southern China and Southeast Asia. Due to its rapid growth, strong salinity tolerance (euryhalinity), and adaptability to captive environments, *L. argentimaculatus* has emerged as a prime candidate for aquaculture, with farming trials already established in regions such as the Philippines^2^. To further enhance aquaculture efficiency and facilitate genetic improvement, there is an urgent need to decipher its genomic information and explore key genetic resources.

However, current genomic resources for *L. argentimaculatus* are insufficient to support system functional analysis, and genomic and transcriptomic data for the entire *Lutjanidae* family remain relatively scarce. Although recent studies have made progress-such as Lai *et al*., who assembled the genome of the closely related crimson snapper (*L. erythropterus*) using long-read sequencing and Hi-C (Contig N50 = 15.13 Mb)^3^, and Shekhar *et al*.^4^, who published a PacBio Sequel II-based assembly for *L. argentimaculatus* (Contig N50 = 12.24 Mb)—these assemblies still exhibit limitations in continuity and precision. Specifically, previous versions contain numerous gaps and fragmented scaffolds, which hinder the resolution of complex genomic regions.

To address these challenges, this study combined PacBio HiFi sequencing with third-generation Oxford Nanopore Technologies (ONT) ultra-long sequencing to generate a gap-free, telomere-to-telomere (T2T) chromosome-level genome assembly for *L. argentimaculatus*. By eliminating gaps and significantly improving contiguity, this high-precision genomic resource will provide critical data support for functional genomics, genetic diversity analysis, and highly efficient selective breeding programs in the future.

## Method

### Institutional review board

All experimental protocols in this study were approved by the Animal Research and Ethics Committee of Guangdong Ocean University Zhanjiang, Guangdong, China (201903003).

### Sample collection and sequencing

An adult female *L. argentimaculatus* was purchased from the Xiashan Seafood Wholesale Market (Zhanjiang, Guangdong Province, China) on January 3, 2024. Prior to tissue sampling, the fish was euthanized via an overdose immersion of tricaine methanesulfonate (MS-222). Muscle tissue was collected for high-molecular-weight genomic DNA extraction. Additionally, total RNA was extracted from eleven representative tissues including blood, liver, muscle, skin, heart, kidney, brain, retina, spleen, gonad and gills to support genome annotation. Blood samples were immediately collected and processed for Hi-C cross-linking.

For genome sequencing, the concentration and integrity of the extracted DNA were rigorously assessed using a Qubit 3.0 Fluorometer. Large DNA fragments were size selected using the BluePippin system to construct a SMRTbell library. Sequencing was performed on the PacBio Revio platform, generating 49.95 Gb of High-fidelity (HiFi/CCS) reads. To facilitate gap closure and resolve complex regions, the same DNA was used to construct a library for Oxford Nanopore Technologies (ONT) sequencing. Following quality control with Qubit 3.0 and Agilent 2100 Bioanalyzer, the library was sequenced on the Oxford Nanopore PromethION platform, yielding 82.8 Gb of raw ONT data, which included 34.4 Gb of ultra-long reads.

For chromosome-scale scaffolding, a Hi-C library was constructed using the cross-linked blood sample. After library quantification and insert size detection (Agilent 2100), DNA Nanoballs (DNBs) were prepared, and sequencing was conducted on the MGI platform, producing 100.97 Gb of raw Hi-C data. For transcriptome analysis, RNA libraries were constructed from the eleven tissues.

After initial quantification and insert size verification, the RNA libraries were sequenced on the MGI high-throughput sequencing platform to generate paired-end reads. To ensure data quality and reproducibility, raw reads were preprocessed and filtered using SOAPnuke (v2.1.8). Specifically, paired-end reads were discarded if: (1) more than 0.5% of the bases in either read were unrecognized; or (2) more than 50% of the bases in either read were low-quality (Phred quality score *Q* ≤ 20). This rigorous quality control pipeline yielded a total of 109.5 Gb of high-quality clean RNA-seq data, which was used for subsequent transcriptome analysis.

### Genome assembly

**Fig. 1:**
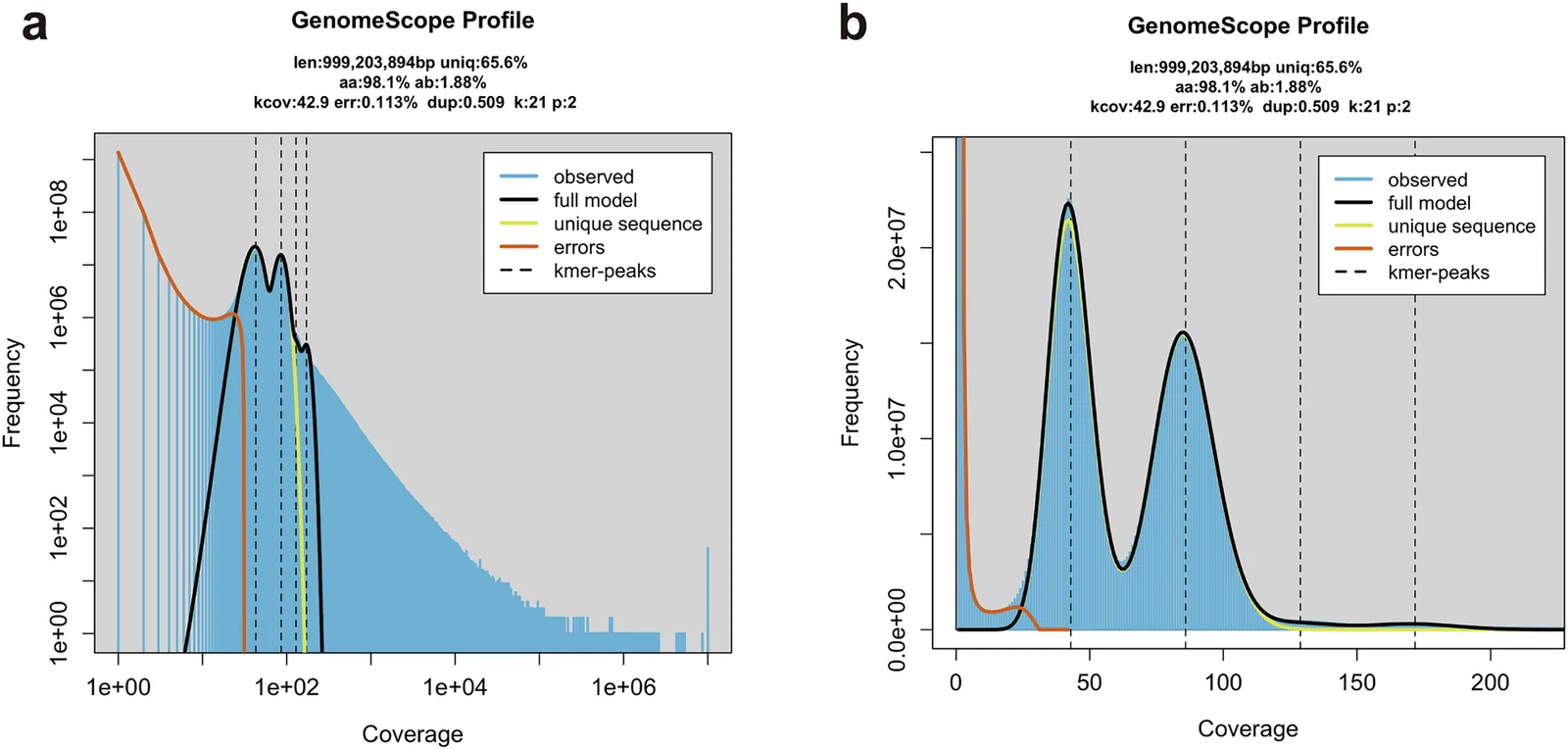
Genome survey and characteristic estimation of *L. argentimaculatus* based on k-mer analysis (k = 21). GenomeScope analysis estimated a genome size of ~952 Mb with 1.88% heterozygosity. (**a**) Log-log plot of k-mer frequency distribution showing observed k-mers (blue), fitted model (black), unique sequences (green dashed), and errors (orange). Vertical dashed lines mark k-mer peaks for heterozygous (~43×) and homozygous (~86×) regions. (**b**) Linear-scale view highlighting the bimodal distribution typical of diploid genomes, with the first peak representing heterozygous k-mers and the second representing homozygous k-mers.

To estimate the genomic characteristics of *L. argentimaculatus*, k-mer analysis was performed using Jellyfish (v2.2.10)^5^ with a k-mer size of 21 (-m 21). The resulting histogram was analyzed using GenomeScope (v2.0)^6^, which estimated the genome size to be approximately 952 Mb with a heterozygosity rate of 1.88% (Fig. 1).

To construct a high-continuity and gap-free foundational assembly for *L. argentimaculatus*, we performed a de novo contig-level assembly using hifiasm (v0.25.0-r726)^7^ Considering the complementarity of different sequencing technologies, we deployed the ONT-hybrid mode (using the–ul–h1–h2 parameter, other parameters use the default settings of hifiasm), which integrated highly accurate PacBio HiFi reads, ultralong Oxford Nanopore (ONT) reads, and Hi-C chromatin conformation data simultaneously. This process yielded a preliminary assembly of ~1.1 Gb, comprising 127 contigs with an N50 of 46.17 Mb. To eliminate contamination and redundancy, the contigs were aligned against the NCBI nucleotide (nt, https://ftp.ncbi.nlm.nih.gov/blast/db/FASTA/nt.gz) and mitochondrial (mt) databases using BLAST (-evalue 1e-5, -perc_identity 0.8, other parameters use the default settings of hifiasm). Following decontamination and self-alignment filtering, the final primary assembly consisted of 118 contigs while maintaining an N50 of 46.17 Mb.

### Chromosome-scale scaffolding and gap-free validation

**Fig. 2:**
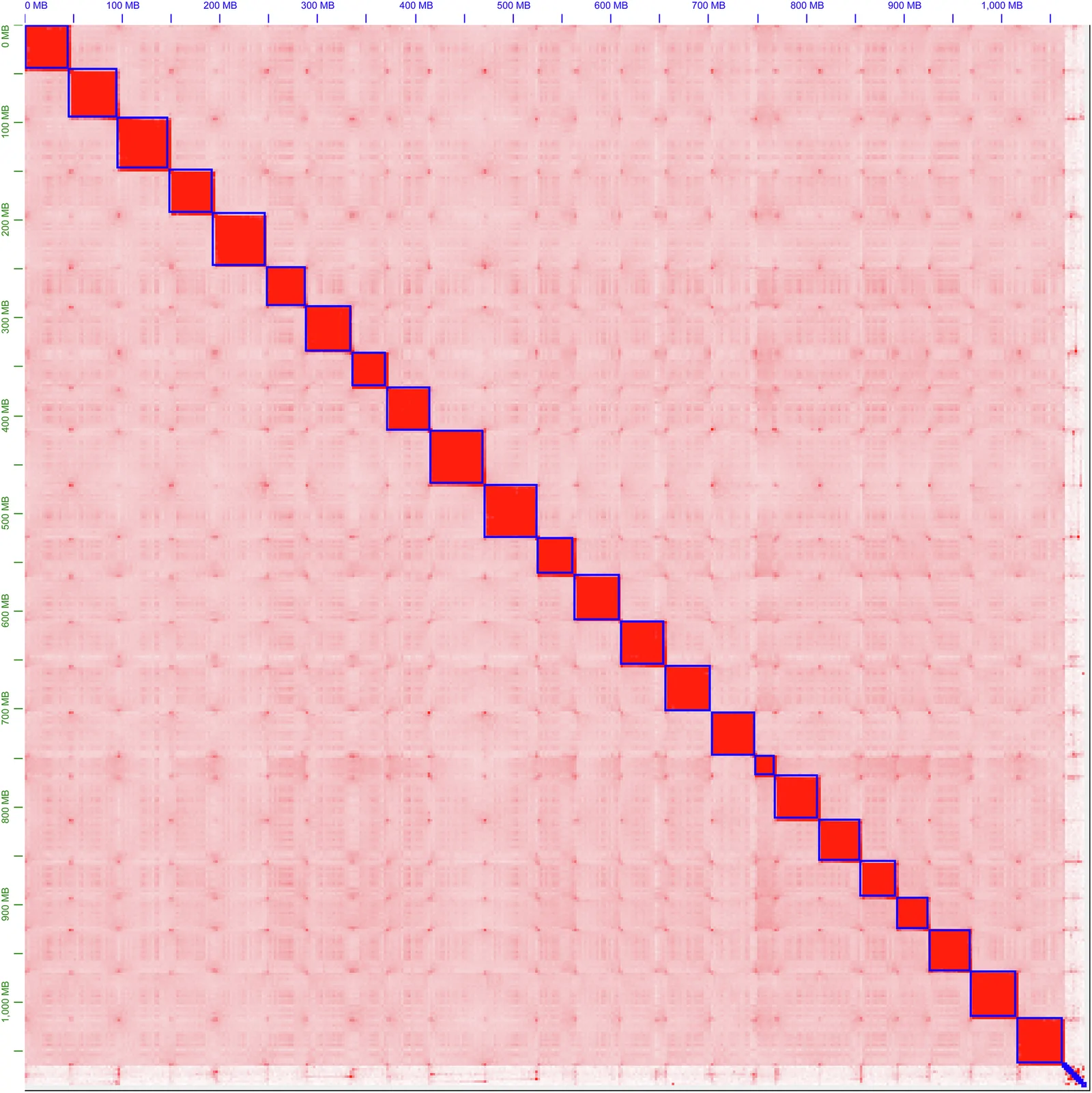
Hi-C contact map of the *L. argentimaculatus* T2T genome assembly. Heatmap showing chromatin interaction frequencies across 24 chromosomes (~1.07 Gb total). Color scale ranges from light pink (low interaction frequency) to dark red (high frequency). Chromosomes are ordered sequentially from chr1 (top-left) to chr24 (bottom-right). Strong diagonal blocks indicate robust intra-chromosomal contacts within each chromosome, confirming successful chromosome-level scaffolding. Minimal off-diagonal signals between chromosomes demonstrate accurate chromosome separation with no inter-chromosomal misassemblies. Clear boundaries between chromosome blocks validate the gap-free, telomere-to-telomere assembly quality. The pattern confirms all 24 contigs correspond to the expected chromosome number of *L. argentimaculatus*.

For chromosome-level scaffolding, Hi-C sequencing data were aligned to the curated contigs using Juicer (v1.6)^8^. The resulting Hi-C contact maps were visually inspected using Juicebox^9^ to correct any potential misassemblies(Fig. 2). The 3d-dna^10^ pipeline was employed to anchor and orient the contigs. The final assembly resulted in 24 scaffolds with an N100 value of 21.43 Mb. Notably, this process produced a gap-free, telomere-to-telomere (T2T) genome assembly for *L. argentimaculatus*. (Tables 1, 2).

**Table 1:** Genome assembly overview statistics.

Shortest (bp)	21438718	N count	0	Gaps	0
Longest (bp)	56436515	N50 (bp)	46217626	Total length (bp)	1066387027
Number	24	N50 numbers	11	Mean length (bp)	44432792.8

**Table 2:** Summary of genome assembly chromosomal characteristics (length and GC content).

Chromosome	length,bp	GC content,%	Chromosome	length,bp	GC content,%
chr1	53619211	0.394128	chr13	45093145	0.396480
chr2	53735898	0.394511	chr14	46249757	0.399617
chr3	56436515	0.396506	chr15	43523598	0.397756
chr4	53075835	0.397488	chr16	43270189	0.397692
chr5	47214804	0.396607	chr17	43217755	0.397481
chr6	48186238	0.395417	chr18	39214184	0.394952
chr7	47608252	0.397034	chr19	40430435	0.399437
chr8	47141593	0.395796	chr20	36260259	0.398365
chr9	46217626	0.397345	chr21	36641815	0.397807
chr10	48989746	0.398604	chr22	32302789	0.399666
chr11	46029471	0.394768	chr23	45148510	0.399967
chr12	45340684	0.398647	chr24	21438718	0.403615

### Telomere identification and synteny analysis

**Fig. 3:**
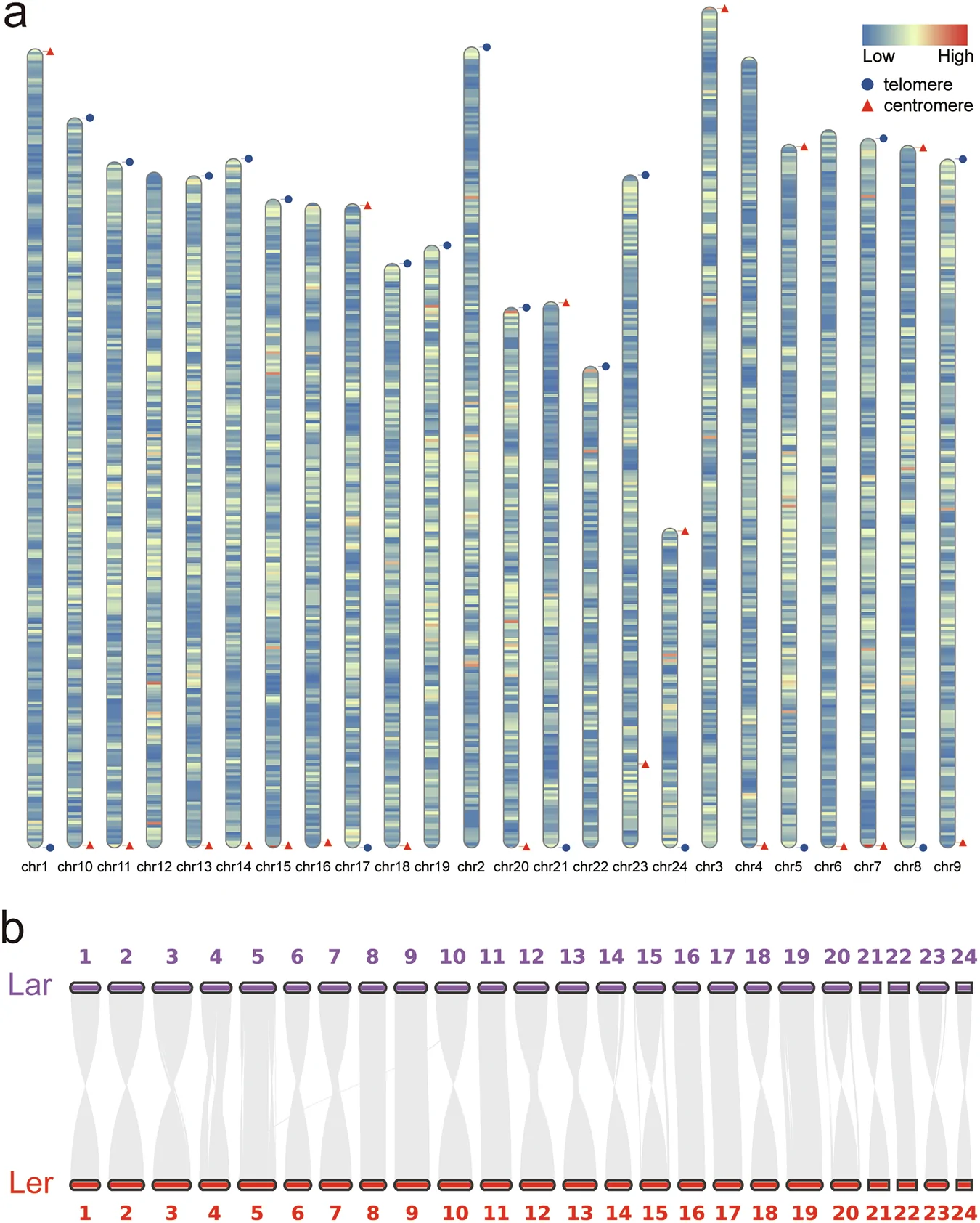
Prediction of chromosomal characteristic and synteny analysis. (**a**) Visualization of telomeres and centromeres across the 24 pseudo-chromosomes of *L. argentimaculatus*. Blue dots represent identified telomeres, and red triangles indicate predicted centromeres. The color gradient of the chromosome bars represents gene density (blue: low; red: high). (**b**) Chromosomal synteny alignment between *L. argentimaculatus* (Lar) and the closely related species *L. erythropterus* (Ler).

Based on the gap-free assembly sequences, telomeric repeats and centromeric regions were identified using quartet (v1.2.0)^11^. The threshold for telomere detection was constrained by the default parameters of the teloexplorer module (-c animal). For centromere prediction via the centrominer module, candidate regions were evaluated based on the density of predicted tandem repeat sequences. To ensure high-confidence annotations, candidate centromere regions with a subcoverage of less than 10% were stringently discarded. Consequently, a total of 19 single-end telomeres and 20 centromeres were finalized (Fig. 3a). This under-prediction is primarily due to the specific genomic architecture of *L. argentimaculatus* as a telocentric species. The co-localization of centromeric and telomeric sequences at the chromosome tips creates extreme regional complexity, posing severe bioinformatic difficulties in completely identifying and distinguishing both feature sequences. Notably, all identified centromeres were located exclusively at the terminal ends of the chromosomes. This genomic architecture is fully consistent with the previously reported karyotype of *L. argentimaculatus*,which is characterized as a terminal-centromere (telocentric) species^12^, thereby validating the high accuracy and reproducibility of our structural annotations. Subsequently, chromosomal identities were validated through synteny analysis against the *L. erythropterus* reference genome using JCVI (v1.5.7)^13^ (Fig. 3b).

### Genome annotation

Repetitive elements were identified and masked prior to gene prediction. A repeat library was constructed using RepeatMasker (v4.1.8)^14^. The genome was then screened against this library using RepeatMasker (-xsmall) to generate a soft-masked genome. In total, 435.37 Mb of repetitive sequences were identified, accounting for 40.83% of the assembled genome.

Protein-coding genes were predicted using a combination of transcript-based, homology-based, and evidence integrated by EVidenceModeler (EVM v2.1.0)^15^. RNA-seq reads were aligned to the soft-masked genome using Hisat2 (v2.2.1)^16^, followed by transcript assembly using Trinity (v2.15.1)^17^ and stringtie (v2.2.1)^18^. PASA (v2.5.3)^19^ was employed to refine gene structures, identify alternative splicing isoforms and annotate UTRs based on the Trinity assemblies. Protein sequences from closely related species (*Danio rerio*, *Oryzias latipes*, *Pagrus major*, and *Lutjanus erythropterus*) were aligned to the genome using miniprot (v0.12-r237)^20^. Gene models were predicted *ab initio* using AUGUSTUS (v3.5.0)^21^ and GeneMark-ETP^22^.

The final gene set was generated by integrating these evidences using EVM with specific weights: PASA-assembled transcripts (weight = 10), stringtie assemblies (weight = 8), TransDecoder (https://github.com/TransDecoder/TransDecoder) predictions derived from PASA (weight = 5), and homologous proteins (weight = 2). Finally, PASA was used again to update the gene models with alternative splicing variations and UTR annotations.

Predicted protein sequences were functionally annotated by aligning them against public databases. Homology searches were performed against the Swiss-Prot, nr (Non-Redundant), and KEGG databases using blastp (e-value < 1e-5)^23^. Protein domains and Gene Ontology (GO) terms were assigned using InterProScan (referencing InterPro, GO, and Pfam databases)^24^, while pathway annotations were performed using eggNOG-mapper^25^.

**Fig. 4:**
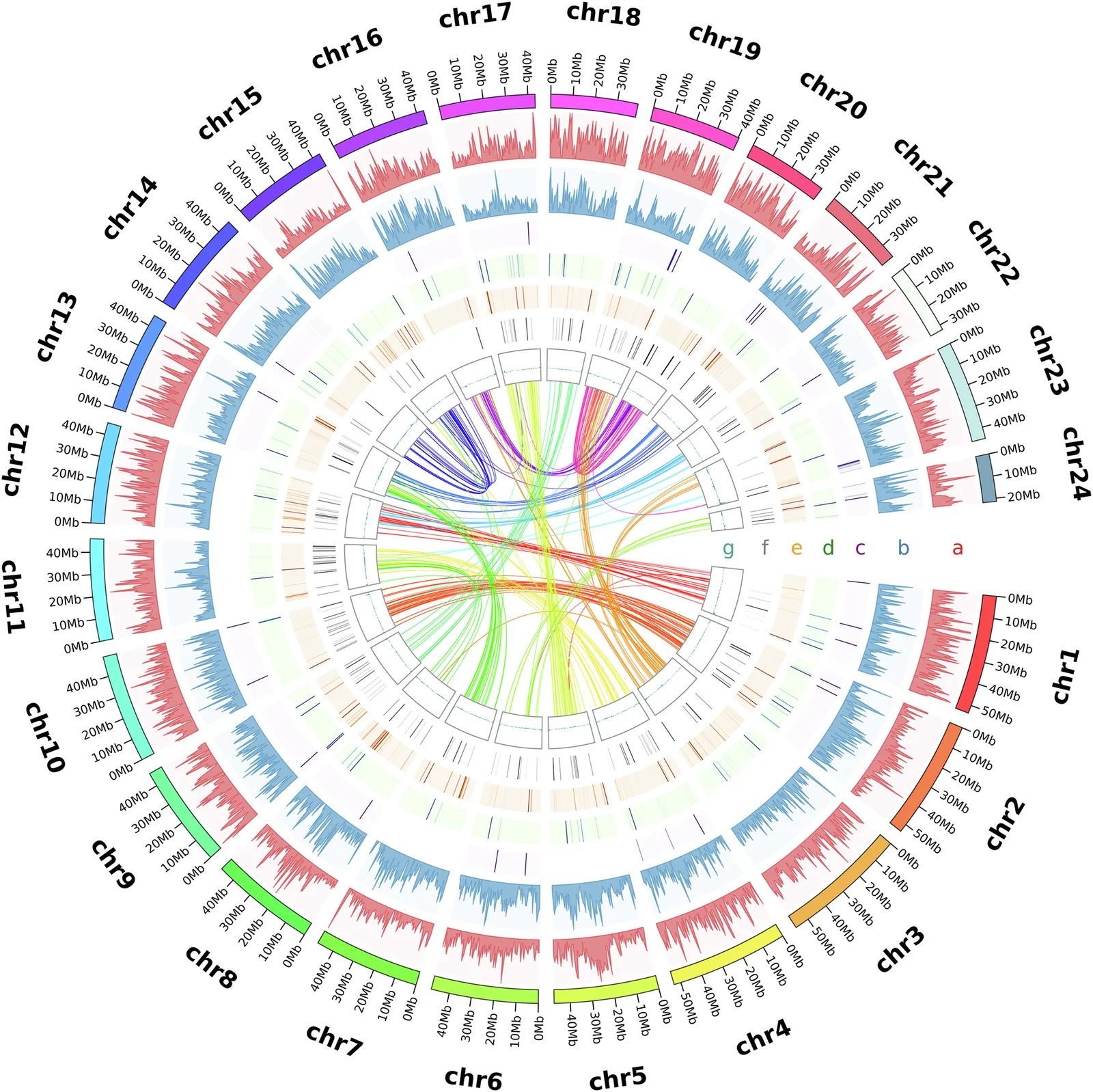
Circos plot of *L. argentimaculatus*. The distribution of functional elements is visualized across the 24 chromosomes. Tracks are arranged from the outer to the inner circles as follows: (**a**) Density of protein-coding genes on the forward strand (red); (**b**) Density of protein-coding genes on the reverse strand (blue); (**c**) Distribution of rRNA (purple); (**d**) Distribution of snRNA (cyan); (**e**) Distribution of tRNA (orange); (**f**) Distribution of miRNA (grey); (**g**) GC content deviation (green). Inner connecting lines represent intra-genomic synteny blocks.

Non-coding RNAs (ncRNAs) were identified using specific tools: tRNAs were predicted using tRNAscan-SE^26^, while miRNAs and snRNAs were annotated using Infernal based on the Rfam database(Fig. 4)^27^.

In total, 23,167 protein-coding genes were predicted in the *L. argentimaculatus* genome. Among them, 22,067 genes were successfully assigned functional annotations. (Table 3).

**Table 3:** Annotation statistics of *L. argentimaculatus*.

Database	Number	Percent
eggNOG	19302	0.833168
interPro	21413	0.924289
NCBI nucleotide	20649	0.891311
SWISS-PROT	17254	0.744766
TrEMBL	20590	0.888764
total	22067	0.952519

## Data Records

The raw sequencing datasets generated during the current study are available in the NCBI Sequence Read Archive (SRA). The genomic DNA sequencing data, comprising PacBio HiFi, ONT and Hi-C reads, have been deposited under SRA Study accession SRP661206^28^. The RNA-seq data derived from eleven tissues are available under SRA Study accession SRP661285^29^.

The final genome assembly has been deposited at NCBI GenBank under the assembly accession GCA_059048685.1^30^. Additionally, the genome assembly file, along with genome annotation files (GFF3, protein sequences, and CDS), has been deposited in the Figshare repository under the https://doi.org/10.6084/m9.figshare.31044799^31^. A detailed summary of accession numbers for all specific sample libraries and sequencing datasets is provided in Table 4.

**Table 4:** Accession numbers of raw sequencing data and repository details for *L. argentimaculatus* genome assembly.

Data Category	Tissue Source	Data Size (Gb)	DataBase	SRA Accession
PacBio HiFi (CCS)	Muscle	49.95	NCBI	SRR36766297^28^
ONT Ultra-long	Muscle	99.56	NCBI	SRR36766299^28^
Hi-C (Arima)	Blood	100.97	NCBI	SRR36766298^28^
Transcriptomic RNA	Blood	10.88	NCBI	SRR36777499^29^
	Brain	11.95	NCBI	SRR36777498^29^
	Gills	11.01	NCBI	SRR36777496^29^
	Gonad	8.40	NCBI	SRR36777495^29^
	Heart	10.26	NCBI	SRR36777494^29^
	Kidney	9.99	NCBI	SRR36777493^29^
	Liver	10.70	NCBI	SRR36777492^29^
	Muscle	8.91	NCBI	SRR36777491^29^
	Retina	8.87	NCBI	SRR36777490^29^
	Skin	9.26	NCBI	SRR36777489^29^
	Spleen	9.27	NCBI	SRR36777497^29^
Genome Assembly	NCBI		GCA_059048685.1^30^	

## Technical Validation

### Genome completeness and continuity assessment

**Fig. 5:**
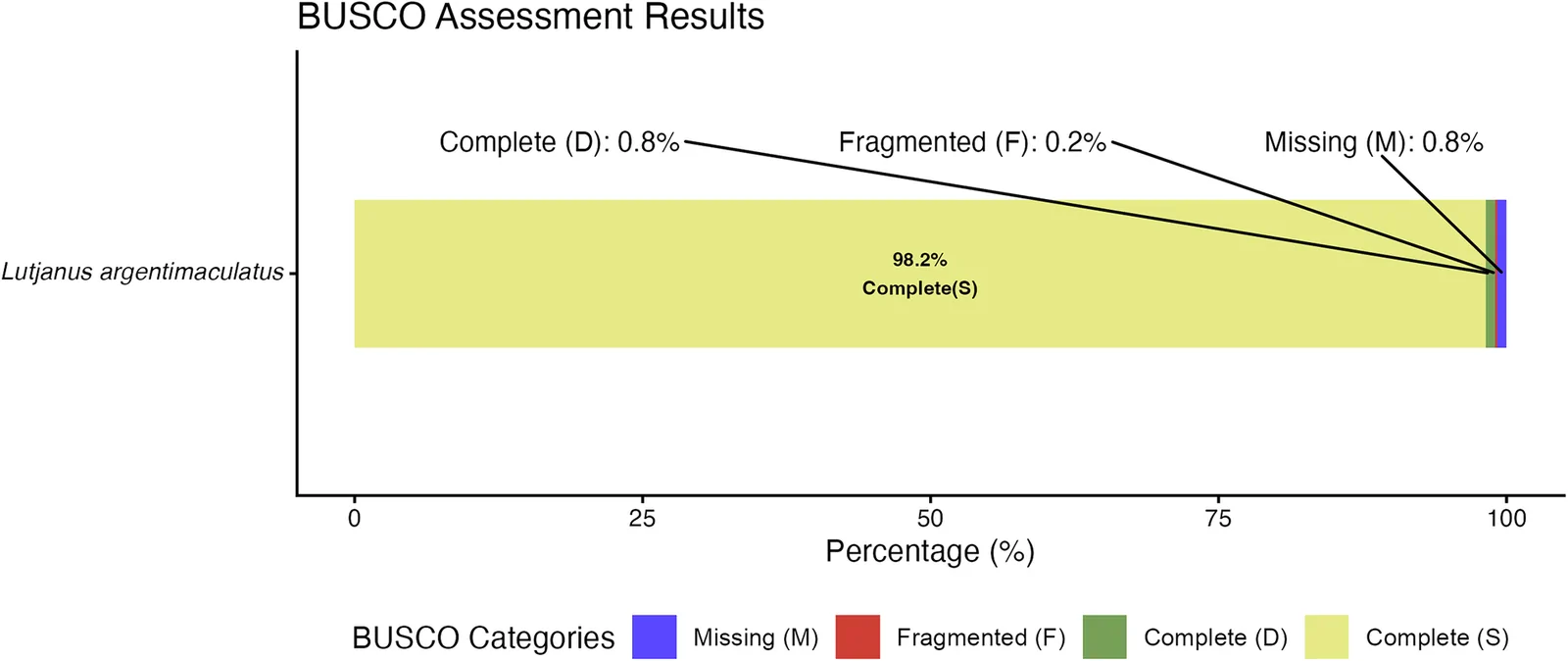
Assessment of genome completeness using BUSCO.

The completeness of the gene space was assessed using BUSCO (v5.8.3)^32^ against the actinopterygii_odb10 lineage dataset. The analysis identified 98.2% of complete BUSCOs as single-copy orthologs, indicating a high level of gene completeness (Fig. 5). Genomic continuity was evaluated using the Long Terminal Repeat Assembly Index (LAI)^33^, which yielded a score of 8.72, reflecting high assembly quality. Furthermore, to verify assembly accuracy, PacBio HiFi reads were mapped back to the assembled genome, resulting in a high mapping rate of 99.88%. Alongside this, ultra-long ONT reads were aligned to the reference genome to confirm continuous coverage and uniform sequencing depth across all 24 chromosomes (Supplementary Figure 1).

### Comparative assessment with existing assemblies

Previous efforts to assemble the *L. campechanus* genome by Norrell *et al*.^34^ resulted in a fragmented assembly with 76,321 contigs. In contrast, the *L. erythropterus* genome assembled by Lai *et al*.^3^ showed better metrics with a contig N50 of 15.14 Mb.

Most recently, Shekhar *et al*.^4^ published a reference genome for *L. argentimaculatus*. While this work established a genomic foundation for the species, it relied on PacBio CLR technology, which is limited by higher error rates. Although scaffolded to the chromosome level using Hi-C, the Shekhar assembly comprises 400 scaffolds and has a contig N50 of only 12.24 Mb, indicating significant residual fragmentation.

In comparison, this study utilized a hybrid strategy combining PacBio HiFi and ONT Ultra-long reads. The ultra-long reads effectively spanned complex repetitive regions, enabling the construction of a gap-free, T2T level assembly. Our assembly consists of exactly 24 contigs corresponding to the 24 chromosomes, with a contig N50 of 46.17 Mb—nearly four times that of the previous version. Additionally, the BUSCO completeness score of 98.2% surpasses the 97.2% reported previously, confirming that this assembly represents the highest quality genomic resource for *L. argentimaculatus* currently available. (Table 5).

**Table 5:** Comparison of genome assembly statistics between this study and the previously reported *L. argentimaculatus* genome.

Metric	Shekhar *et al*.^4^	This Study
**Assembly Level**	Chromosome (Gapped)	Chromosome (Gap-free)
**Contig N50**	12.24 Mb	46.17 Mb
**Scaffold N50**	33.80 Mb	46.17 Mb
**Number of Scaffolds**	400	24
**Number of Contigs**	699	24
**Gaps**	Present	0
**BUSCO Completeness**	97.20%	98.20%

## Supplementary information

Supplementary Figure 1

## Data Availability

All data processing commands and pipelines were carried out in accordance with the instructions and guidelines provided by the relevant bioinformatics software.

## References

[1] Tiralongo, F., Giovos, I., Doumpas, N. Is the mangrove red snapper *Lutjanus argentimaculatus* (forsskål, 1775) established in the eastern Mediterranean sea? First records from Greece through a citizen science project. in *Bioinvasions Records* 617–626 (2019).

[2] Emata, A. C. Reproductive performance in induced and spontaneous spawning of the mangrove red snapper, *Lutjanus argentimaculatus*: a potential candidate species for sustainable aquaculture. *Aquac Res* **34**, 849–857 (2003).

[3] Lai, Z. *et al*. The first high-quality chromosome-level genome of the *Lutjanus erythropterus* (Bloch, 1790) using single-tube long fragment reads and hi-C technologies. *Genome Biol Evol* **15**, evad171 (2023). 37768150 10.1093/gbe/evad171 PMC10558211

[4] Shekhar, M. S. *et al*. Genome assembly, Full-length transcriptome, and isoform diversity of Red Snapper, *Lutjanus argentimaculatus*. *Sci Data* **11**, 796 (2024). 39025998 10.1038/s41597-024-03633-1 PMC11258364

[5] Marçais, G. & Kingsford, C. A fast, lock-free approach for efficient parallel counting of occurrences of k-mers. *Bioinformatics* **27**, 764–770 (2011). 21217122 10.1093/bioinformatics/btr011 PMC3051319

[6] Vurture, G. W. *et al*. GenomeScope: fast reference-free genome profiling from short reads. *Bioinformatics* **33**, 2202–2204 (2017). 28369201 10.1093/bioinformatics/btx153 PMC5870704

[7] Cheng, H., Asri, M., Lucas, J., Koren, S. & Li, H. Scalable telomere-to-telomere assembly for diploid and polyploid genomes with double graph. *Nat Methods* **21**, 967–970 (2024). 38730258 10.1038/s41592-024-02269-8 PMC11214949

[8] Durand, N. C. *et al*. Juicer provides a one-click system for analyzing loop-resolution hi-C experiments. *Cell Syst* **3**, 95–98 (2016). 27467249 10.1016/j.cels.2016.07.002 PMC5846465

[9] Dudchenko, O. *et al*. The juicebox assembly tools module facilitates de novo assembly of mammalian genomes with chromosome-length scaffolds for under $1000. *bioRxiv* 10.1101/254797 (2018).

[10] Dudchenko, O. *et al*. De novo assembly of the *Aedes aegypti* genome using hi-C yields chromosome-length scaffolds. *Science* **356**, 92–95 (2017). 28336562 10.1126/science.aal3327 PMC5635820

[11] Lin, Y. *et al*. quarTeT: a telomere-to-telomere toolkit for gap-free genome assembly and centromeric repeat identification. *Hortic Res* **10**, uhad127 (2023). 37560017 10.1093/hr/uhad127 PMC10407605

[12] Cao, F.-J., Li, C.-L., Liu, C.-W. Studies on the Karyotypes of *Lutjanus erythopterus*, *Lutjanus argentimaculatus* and *Lutjanus bohar*. *Marine Sciences* 43–46 (2002).

[13] Tang, H. *et al*. JCVI: a versatile toolkit for comparative genomics analysis. *Imeta* **3**, e211 (2024). 39135687 10.1002/imt2.211 PMC11316928

[14] Flynn, J. M. *et al*. RepeatModeler2 for automated genomic discovery of transposable element families. *Proc Natl Acad Sci USA* **117**, 9451–9457 (2020). 32300014 10.1073/pnas.1921046117 PMC7196820

[15] Haas, B. J. *et al*. Automated eukaryotic gene structure annotation using EVidenceModeler and the program to assemble spliced alignments. *Genome Biol* **9**, R7 (2008). 18190707 10.1186/gb-2008-9-1-r7 PMC2395244

[16] Kim, D., Paggi, J. M., Park, C., Bennett, C. & Salzberg, S. L. Graph-based genome alignment and genotyping with HISAT2 and HISAT-genotype. *Nat Biotechnol* **37**, 907–915 (2019). 31375807 10.1038/s41587-019-0201-4 PMC7605509

[17] Grabherr, M. G. *et al*. Full-length transcriptome assembly from RNA-seq data without a reference genome. *Nat Biotechnol* **29**, 644–652 (2011). 21572440 10.1038/nbt.1883 PMC3571712

[18] Shumate, A., Wong, B., Pertea, G. & Pertea, M. Improved transcriptome assembly using a hybrid of long and short reads with StringTie. *PLoS Comput Biol* **18**, e1009730 (2022). 35648784 10.1371/journal.pcbi.1009730 PMC9191730

[19] Haas, B. J. *et al*. Improving the arabidopsis genome annotation using maximal transcript alignment assemblies. *Nucleic Acids Res* **31**, 5654–5666 (2003). 14500829 10.1093/nar/gkg770 PMC206470

[20] Li, H. Protein-to-genome alignment with miniprot. *Bioinformatics* **39**, btad014 (2023). 36648328 10.1093/bioinformatics/btad014 PMC9869432

[21] Stanke, M., Diekhans, M., Baertsch, R. & Haussler, D. Using native and syntenically mapped cDNA alignments to improve *de novo* gene finding. *Bioinformatics* **24**, 637–644 (2008). 18218656 10.1093/bioinformatics/btn013

[22] Bruna, T., Lomsadze, A. & Borodovsky, M. A new gene finding tool GeneMark-ETP significantly improves the accuracy of automatic annotation of large eukaryotic genomes. *Bioinformatics* 10.1101/2023.01.13.524024 (2023). 38866548 PMC11216313

[23] Camacho, C. *et al*. BLAST+: architecture and applications. *BMC Bioinformatics* **10**, 421 (2009). 20003500 10.1186/1471-2105-10-421 PMC2803857

[24] Jones, P. *et al*. InterProScan 5: genome-scale protein function classification. *Bioinformatics* **30**, 1236–1240 (2014). 24451626 10.1093/bioinformatics/btu031 PMC3998142

[25] Cantalapiedra, C. P., Hernández Plaza, A., Letunic, I., Bork, P. & Huerta Cepas, J. eggNOG-mapper v2: Functional Annotation, Orthology Assignments, and Domain Prediction at the Metagenomic Scale. *Mol Biol Evol* **38**, 5825–5829 (2021). 34597405 10.1093/molbev/msab293 PMC8662613

[26] tRNAscan-SE 2.0: improved detection and functional classification of transfer RNA genes | nucleic acids research | oxford academic. 10.1093/nar/gkab688 PMC8450103 34417604

[27] Ontiveros Palacios, N. *et al*. Rfam 15: RNA families database in 2025. *Nucleic Acids Res* **53**, D258–D267 (2025). 39526405 10.1093/nar/gkae1023 PMC11701678

[28] *NCBI Sequence Read Archive* https://identifiers.org/ncbi/insdc.sra:SRP661206 (2026).

[29] *NCBI Sequence Read Archive* https://identifiers.org/ncbi/insdc.sra:SRP661285 (2026).

[30] *NCBI GenBank* https://identifiers.org/ncbi/insdc.gca:GCA_059048685.1 (2026).

[31] Xiang, Y., Lu, Z.-C., Jiang, H.-L., Guo, Y.-S. & Wang, Z.-D. A gap-free, telomere-to-telomere chromosome-scale genome assembly of the mangrove red snapper, *Lutjanus argentimaculatus*. *figshare*10.6084/m9.figshare.31044799 (2026).42706272

[32] Simão, F. A., Waterhouse, R. M., Ioannidis, P., Kriventseva, E. V. & Zdobnov, E. M. BUSCO: assessing genome assembly and annotation completeness with single-copy orthologs. *Bioinformatics* **31**, 3210–3212 (2015). 26059717 10.1093/bioinformatics/btv351

[33] Ellinghaus, D., Kurtz, S. & Willhoeft, U. LTRharvest, an efficient and flexible software for de novo detection of LTR retrotransposons. *BMC Bioinformatics* **9**, 18 (2008). 18194517 10.1186/1471-2105-9-18 PMC2253517

[34] Norrell, A. E., Jones, K. L. & Saillant, E. A. Development and characterization of genomic resources for a non-model marine teleost, the red snapper (*lutjanus campechanus*, lutjanidae): construction of a high-density linkage map, anchoring of genome contigs and comparative genomic analysis. *PLoS One* **15**, e0232402 (2020). 32348345 10.1371/journal.pone.0232402 PMC7190162

