# Supplementary material for: A gap-free, telomere-to-telomere chromosome-scale genome assembly of the mangrove red snapper, Lutjanus argentimaculatus
Source: Sci Data. 2026 Aug 5;13:1290. doi: 10.1038/s41597-026-08016-2 (PMC13550438; doi:10.1038/s41597-026-08016-2)
Supplement: Supplementary file 1 [file 41597_2026_8016_MOESM1_ESM.pdf]

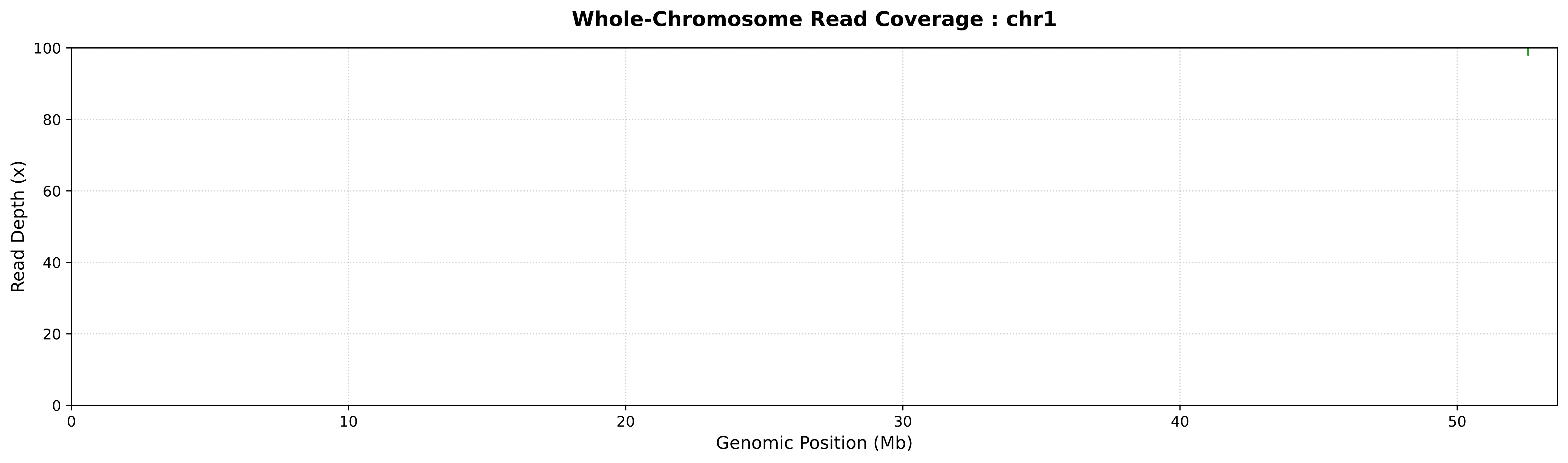
Whole-Chromosome Read Coverage : chr1
Read Depth (x)
Genomic Position (Mb)
0
20
40
60
80
100
0
10
20
30
40
50

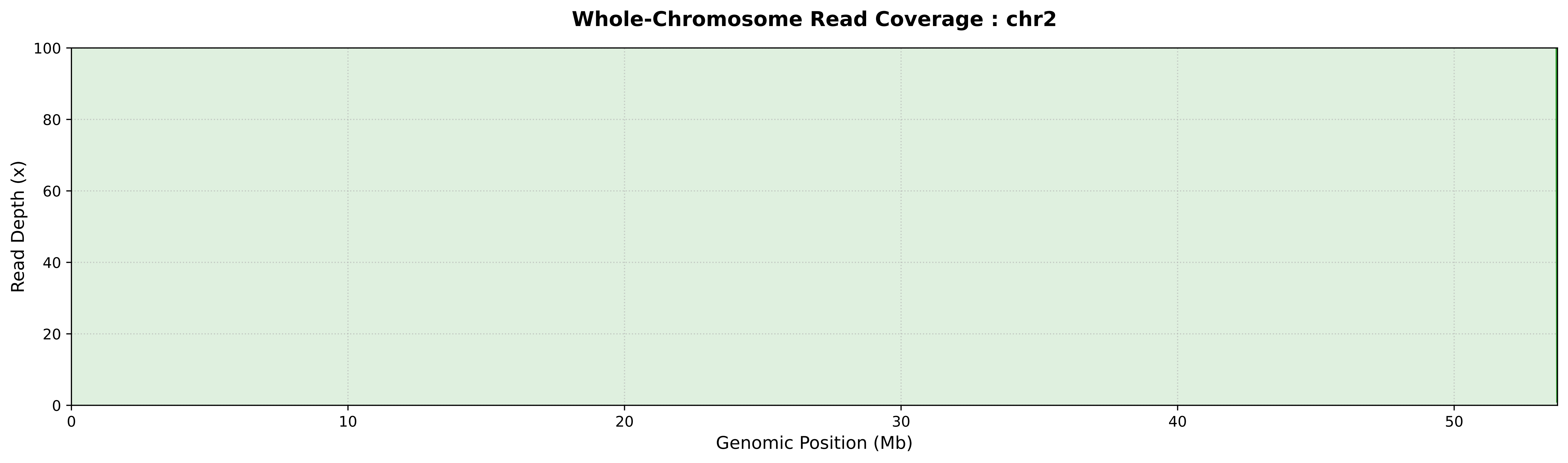

Whole-Chromosome Read Coverage : chr2
Read Depth (x)
100
80
60
40
20
0
10
20
30
40
50
Genomic Position (Mb)

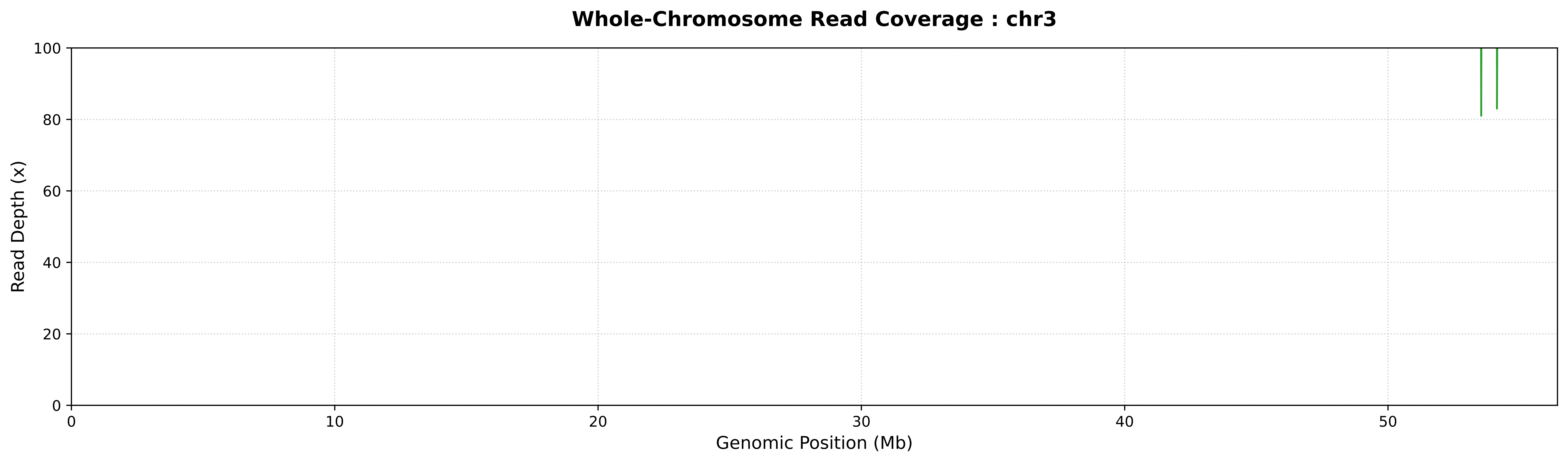

Whole-Chromosome Read Coverage : chr3
Read Depth (x)
Genomic Position (Mb)
0
10
20
30
40
50
0
20
40
60
80
100

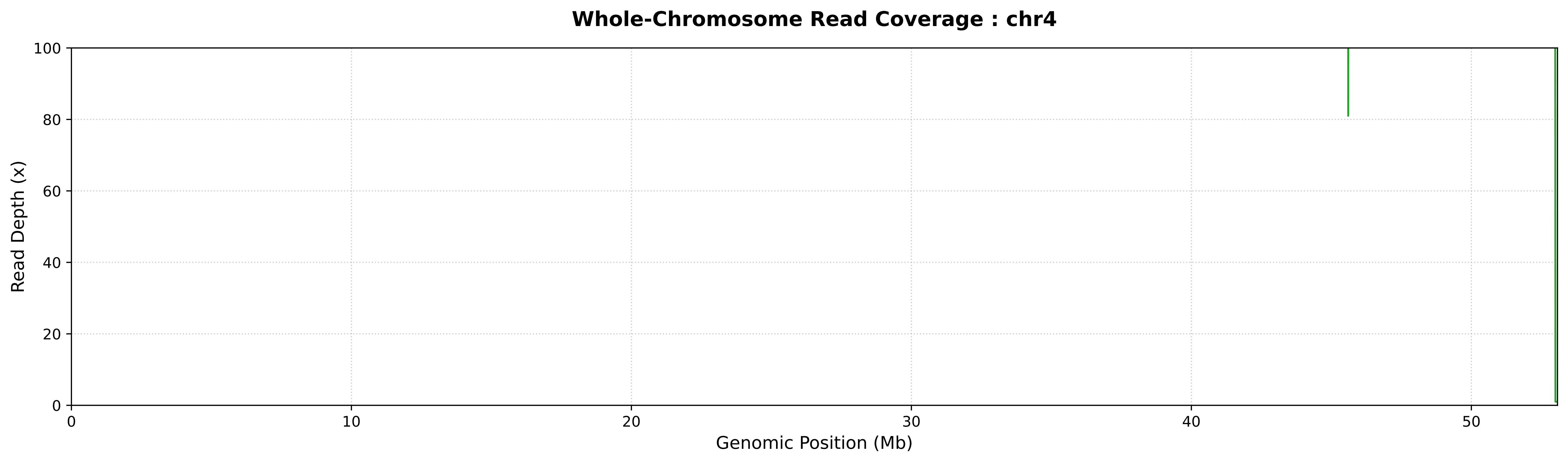
Whole-Chromosome Read Coverage : chr4
Read Depth (x)
0
20
40
60
80
100
0
10
20
30
40
50
Genomic Position (Mb)

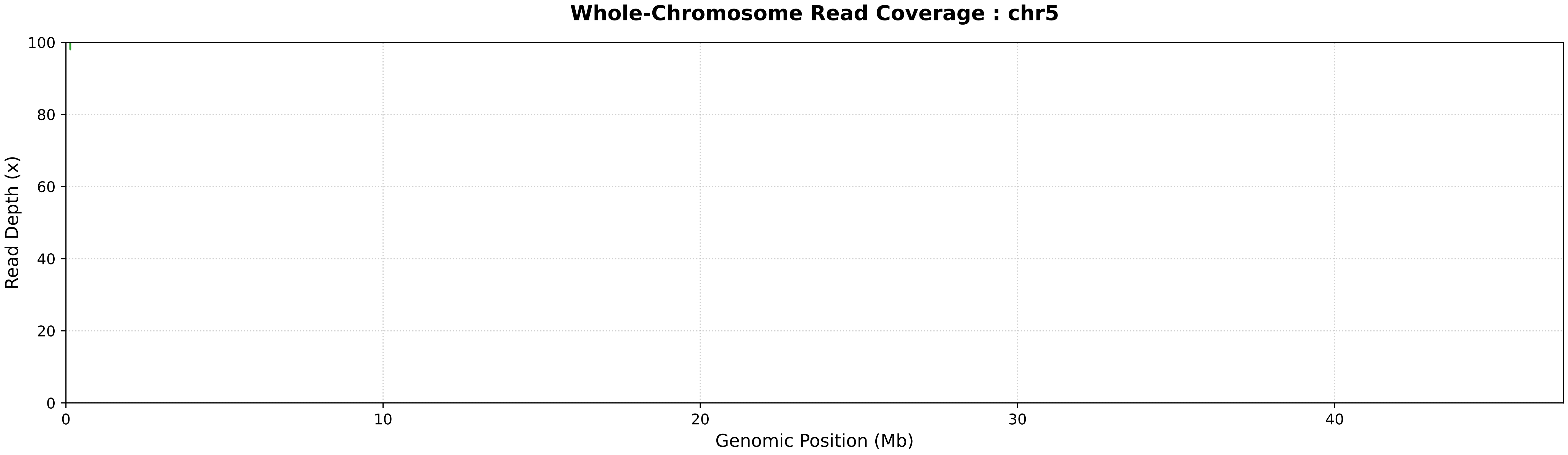

Whole-Chromosome Read Coverage : chr5
Read Depth (x)
Genomic Position (Mb)
0
20
40
60
80
100
0
10
20
30
40

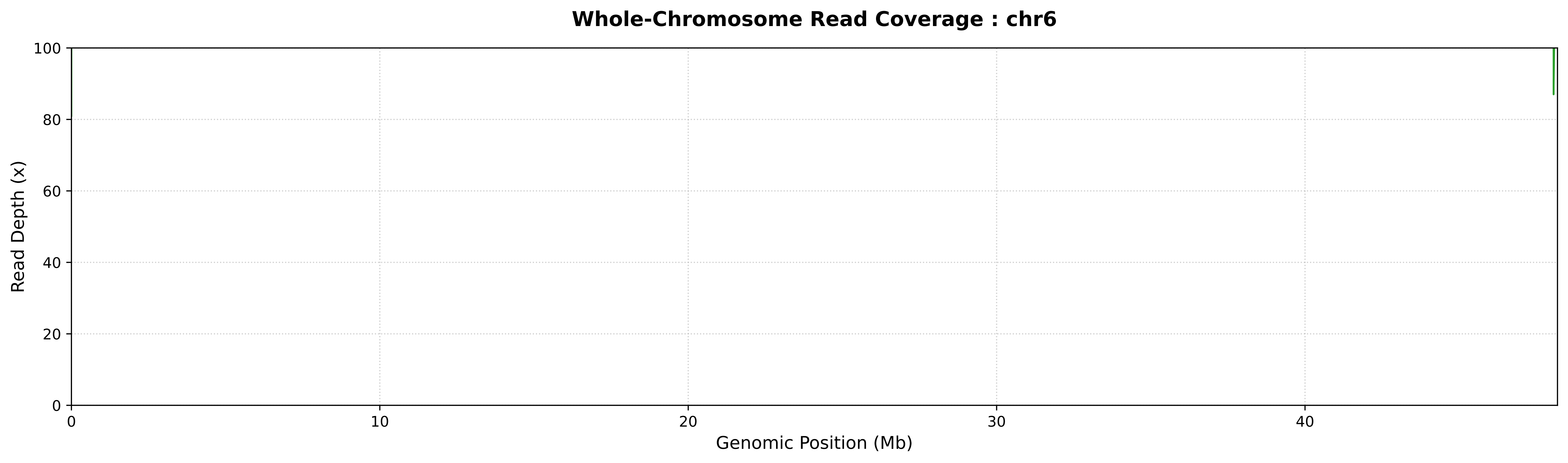
Whole-Chromosome Read Coverage : chr6
Read Depth (x)
Genomic Position (Mb)
0
20
40
60
80
100
0
10
20
30
40

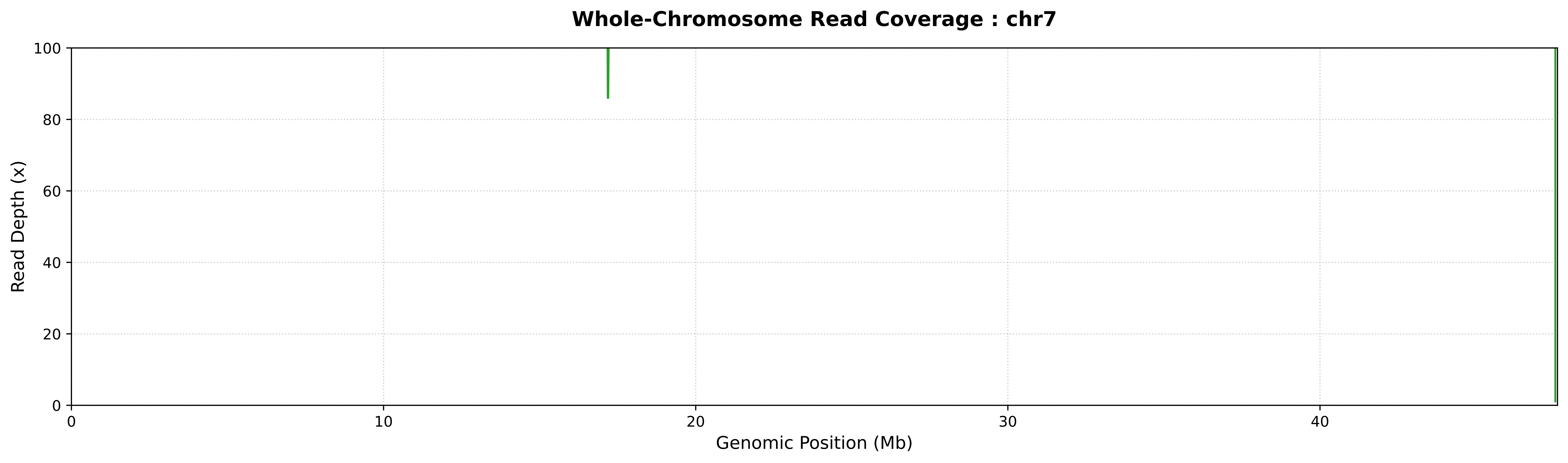
Whole-Chromosome Read Coverage : chr7
Read Depth (x)
0
20
40
60
80
100
0
10
20
30
40
Genomic Position (Mb)

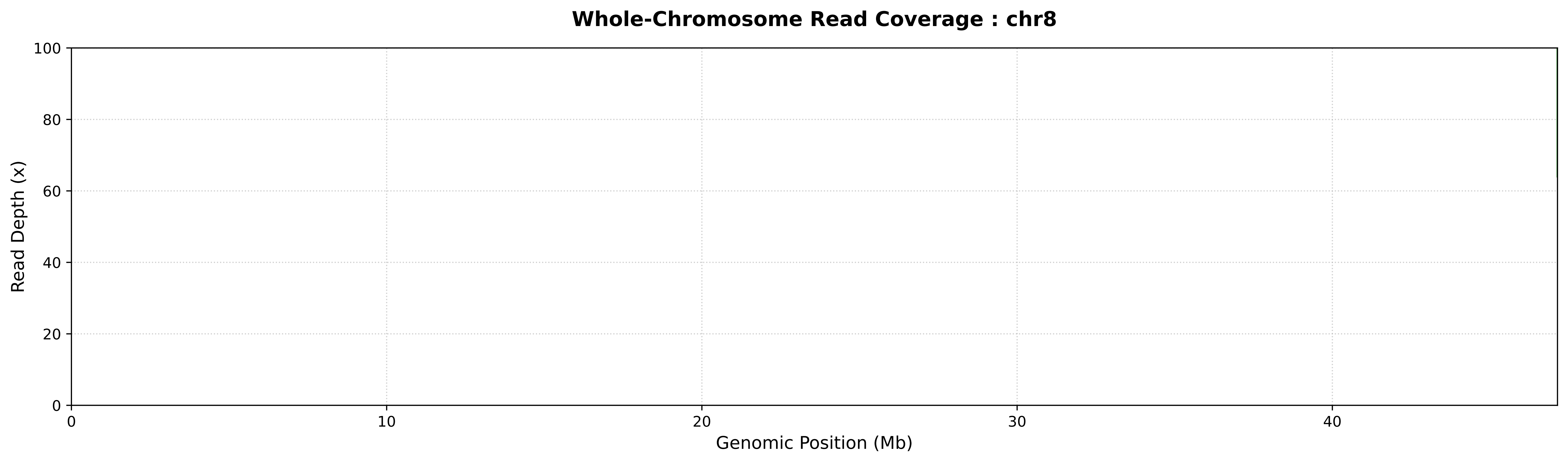
Whole-Chromosome Read Coverage : chr8
Read Depth (x)
Genomic Position (Mb)
0
20
40
60
80
100
0
10
20
30
40

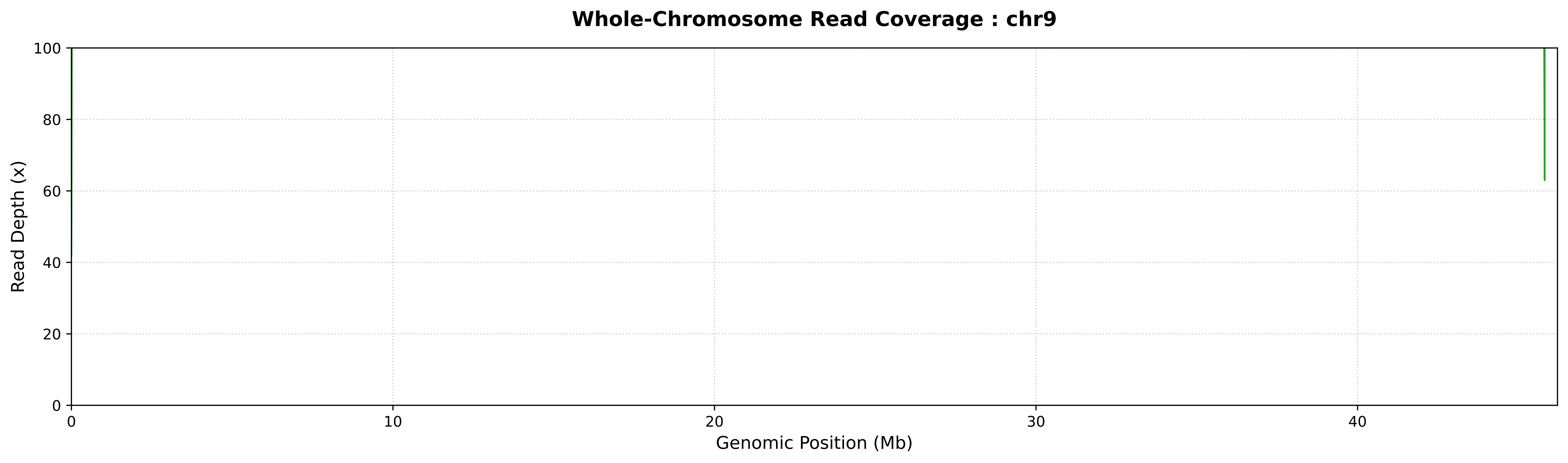
Whole-Chromosome Read Coverage : chr9
Read Depth (x)
0
20
40
60
80
100
Genomic Position (Mb)
0
10
20
30
40

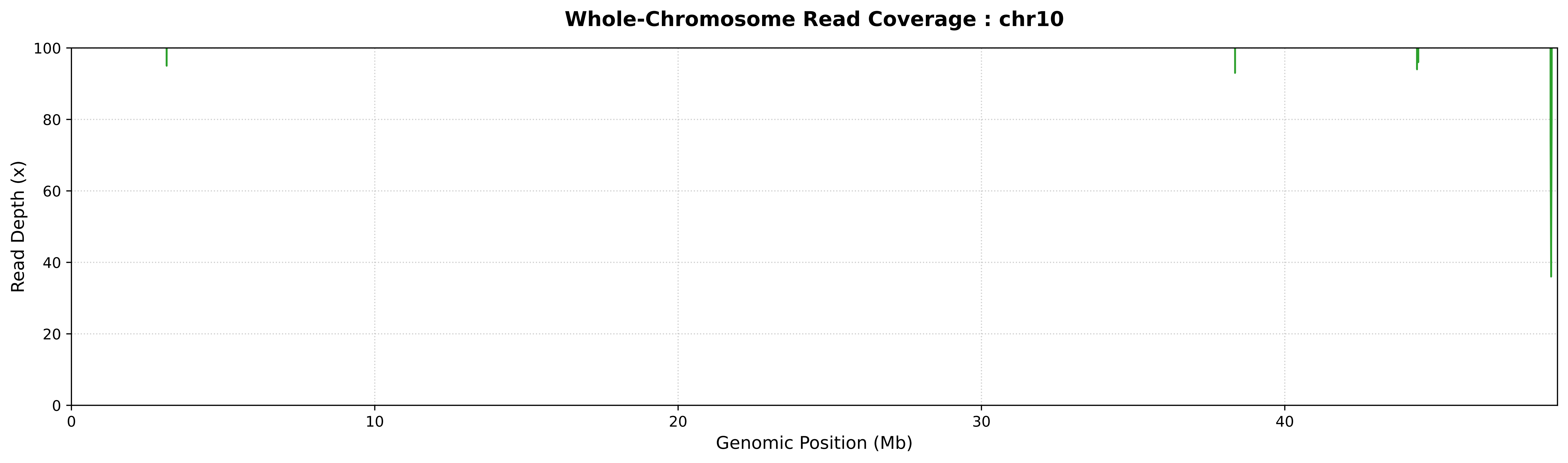
Whole-Chromosome Read Coverage : chr10
Read Depth (x)
Genomic Position (Mb)
0
20
40
60
80
100
0
10
20
30
40

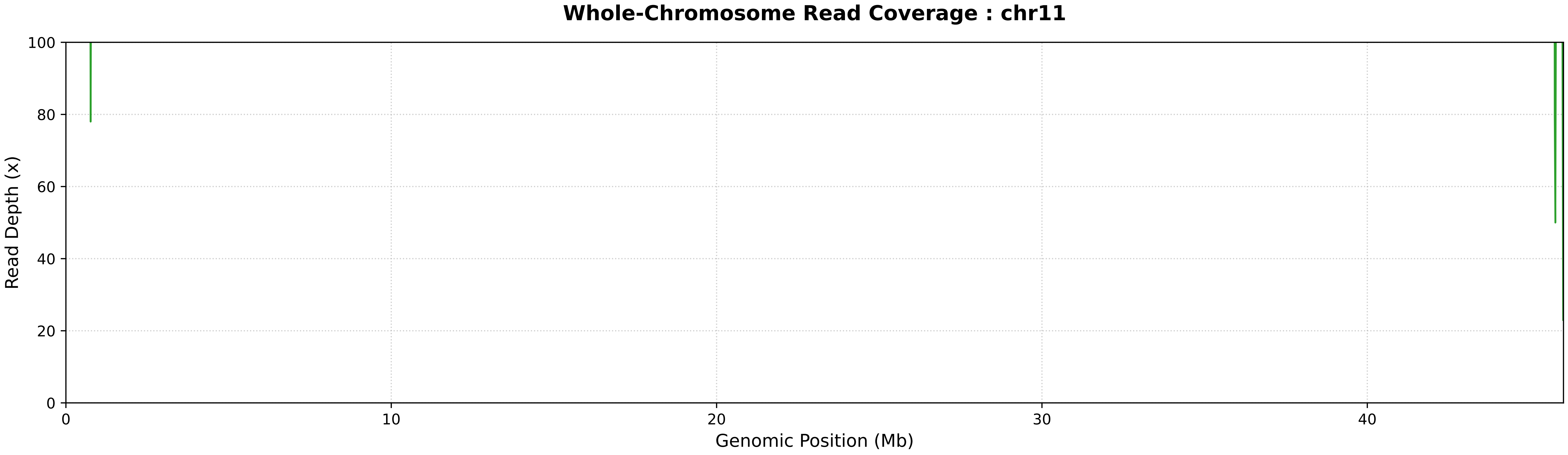
Whole-Chromosome Read Coverage : chr11
Read Depth (x)
Genomic Position (Mb)
0
20
40
60
80
100
0
10
20
30
40

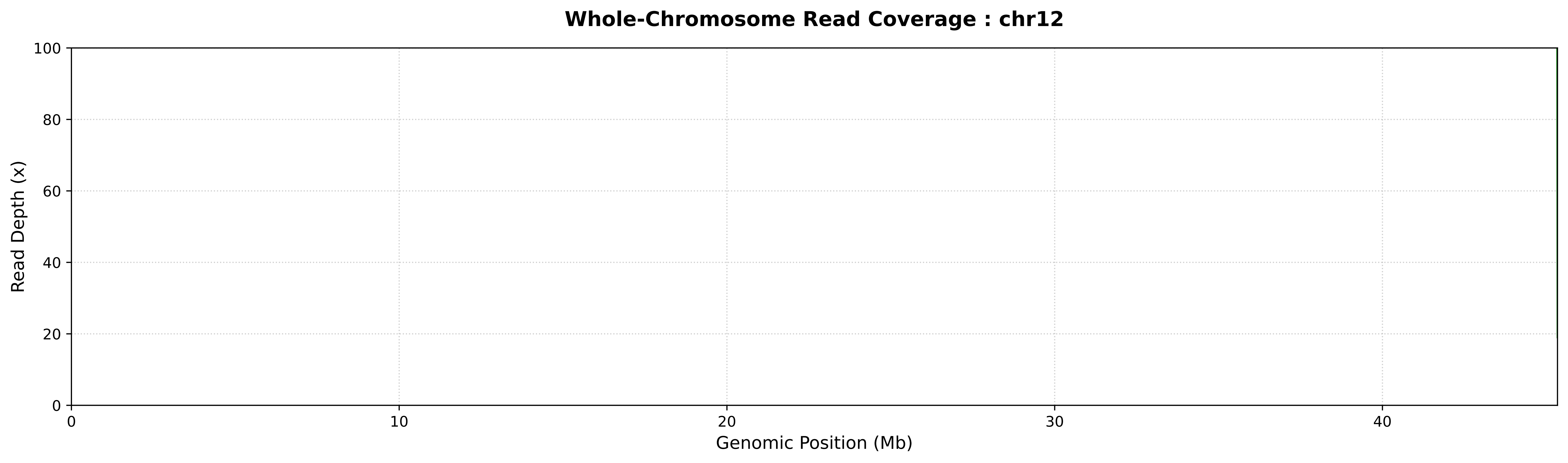
Whole-Chromosome Read Coverage : chr12
Read Depth (x)
Genomic Position (Mb)
0
20
40
60
80
100
0
10
20
30
40

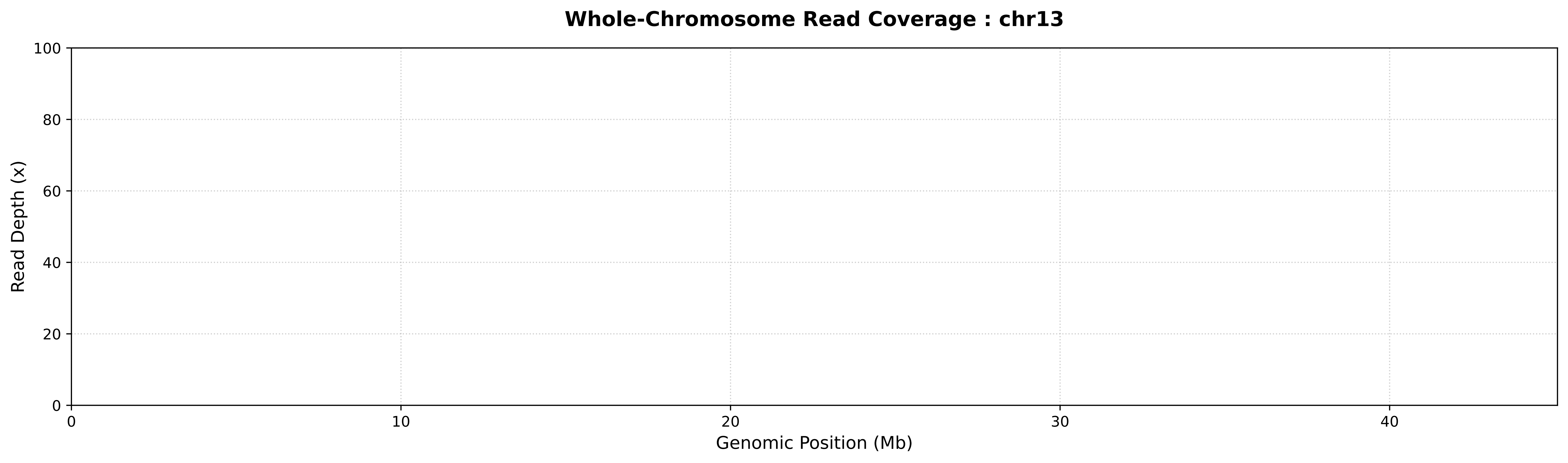
Whole-Chromosome Read Coverage : chr13
Read Depth (x)
0
20
40
60
80
100
0
10
20
30
40
Genomic Position (Mb)

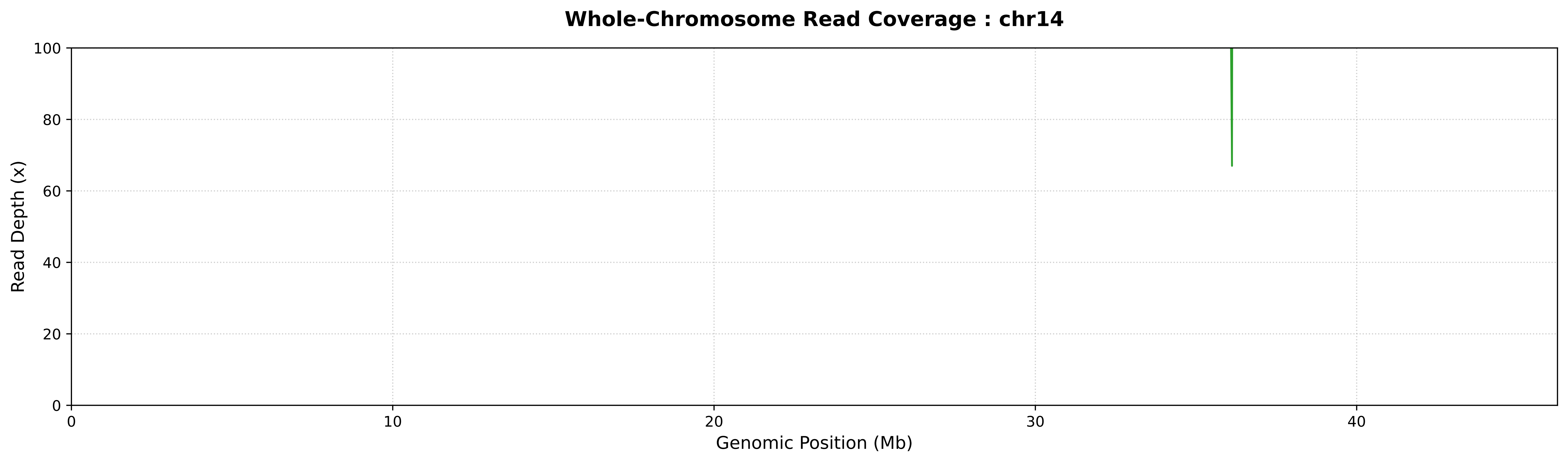
Whole-Chromosome Read Coverage : chr14
Read Depth (x)
Genomic Position (Mb)
0
20
40
60
80
100
0
10
20
30
40

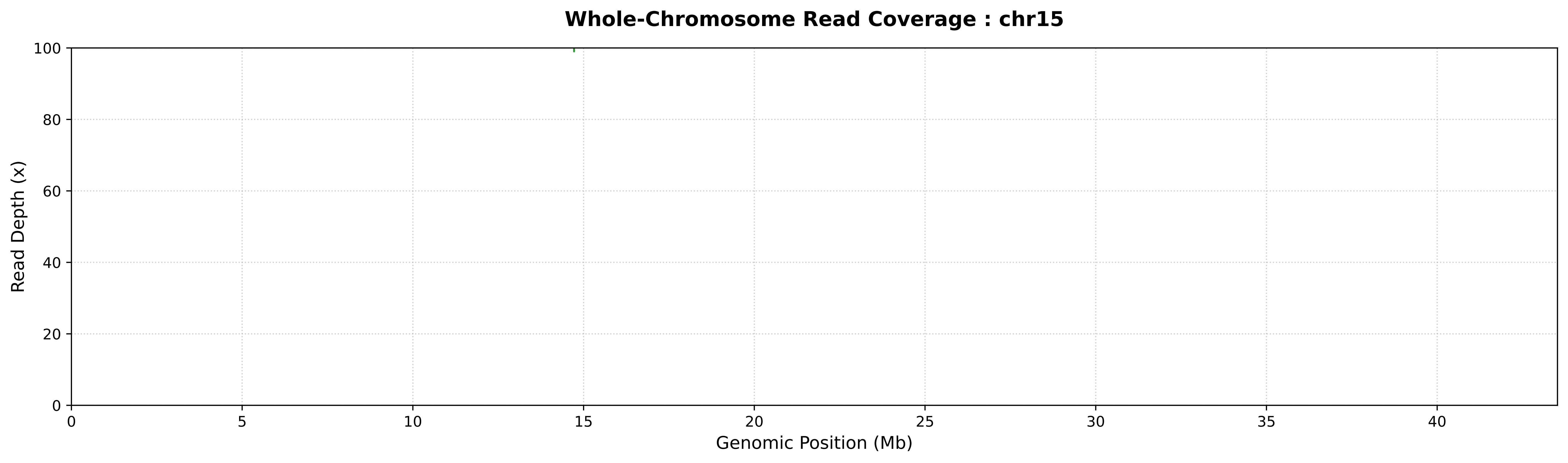
Whole-Chromosome Read Coverage : chr15
Read Depth (x)
100
80
60
40
20
0
0
5
10
15
20
25
30
35
40
Genomic Position (Mb)

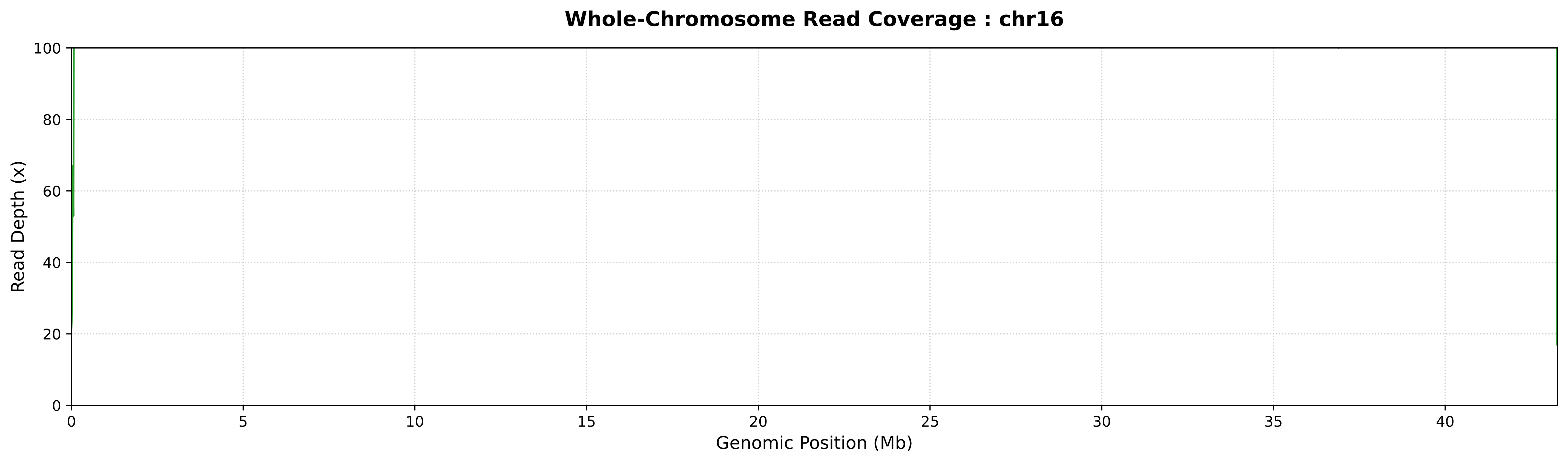

Whole-Chromosome Read Coverage : chr16
Read Depth (x)
0
20
40
60
80
100
Genomic Position (Mb)
0
5
10
15
20
25
30
35
40

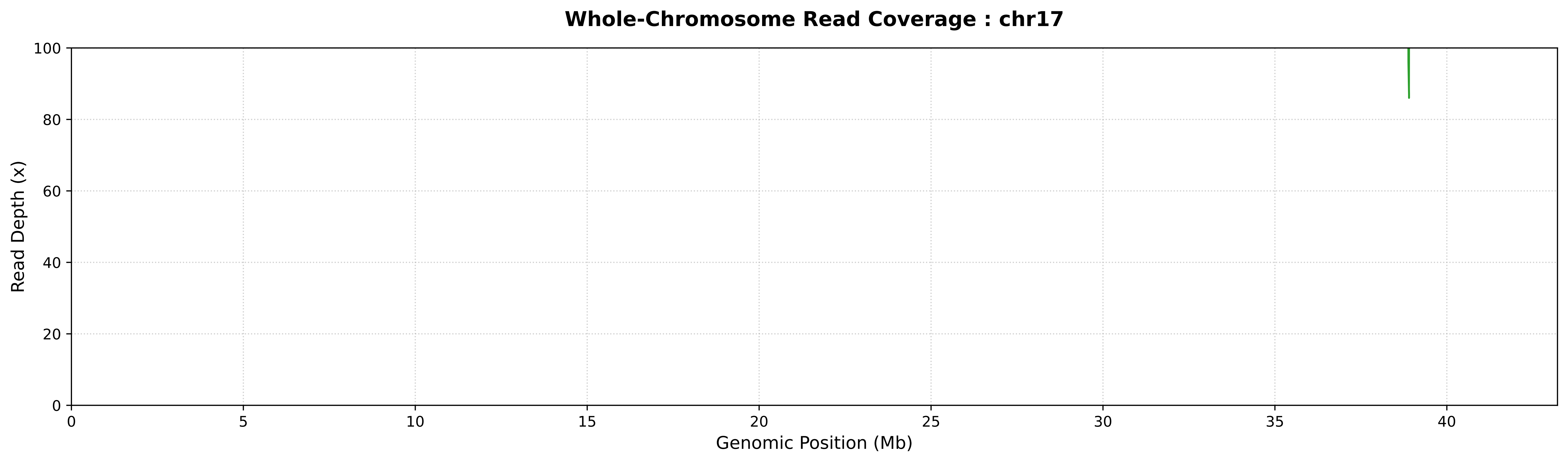

Whole-Chromosome Read Coverage : chr17
Read Depth (x)
0
20
40
60
80
100
Genomic Position (Mb)
0
5
10
15
20
25
30
35
40

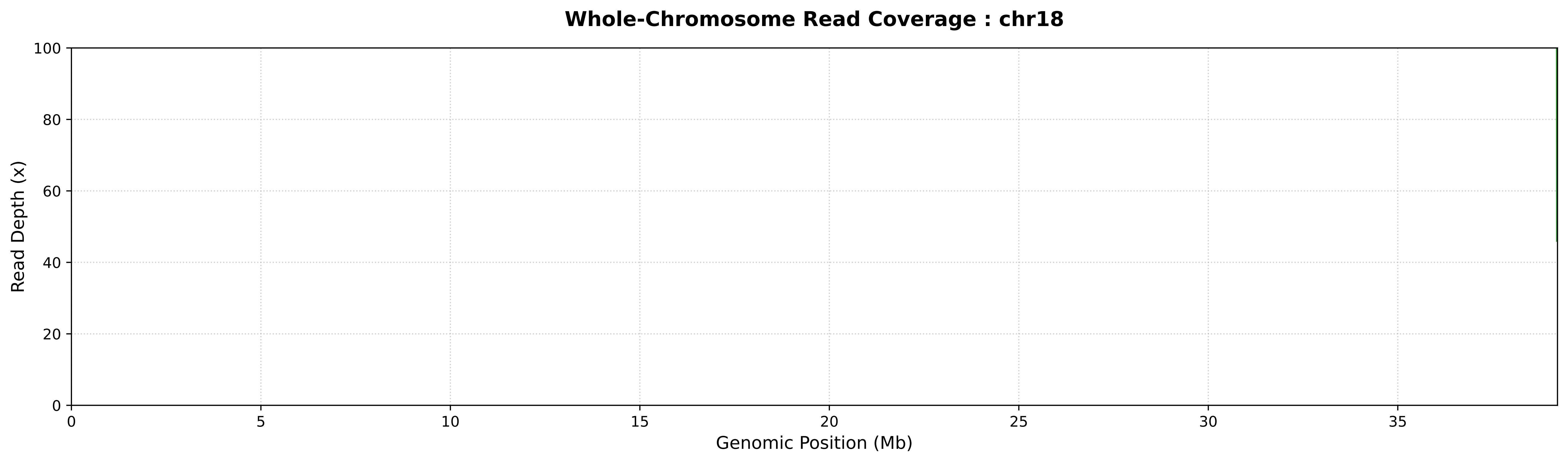
Whole-Chromosome Read Coverage : chr18
Read Depth (x)
Genomic Position (Mb)
0
20
40
60
80
100
0
5
10
15
20
25
30
35

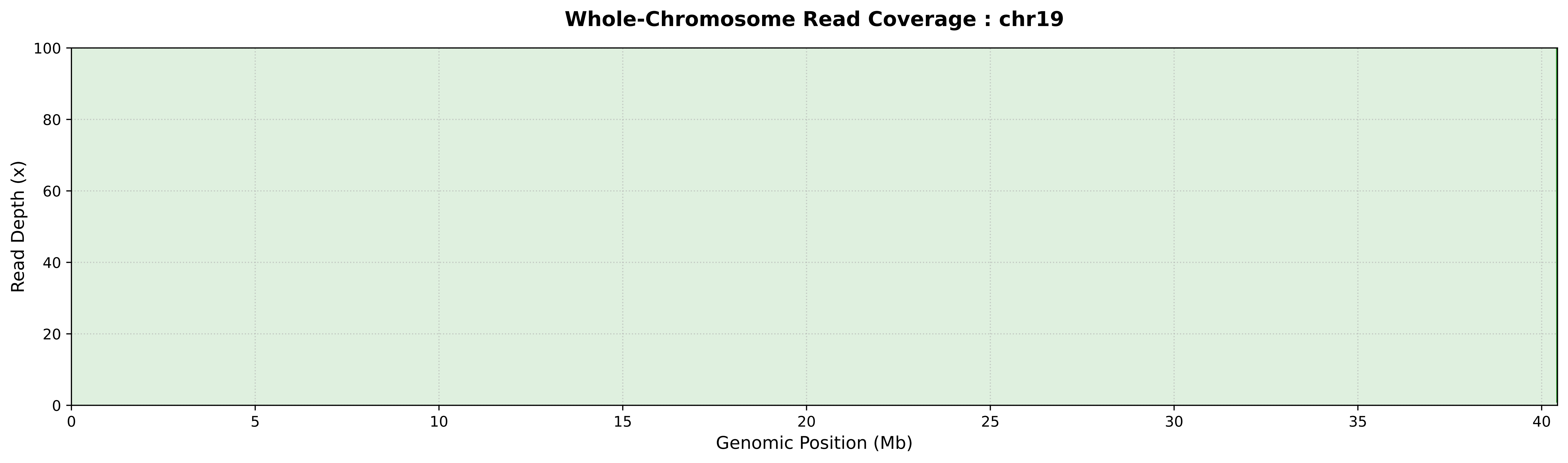
Whole-Chromosome Read Coverage : chr19
Read Depth (x)
Genomic Position (Mb)
0
20
40
60
80
100
0
5
10
15
20
25
30
35
40

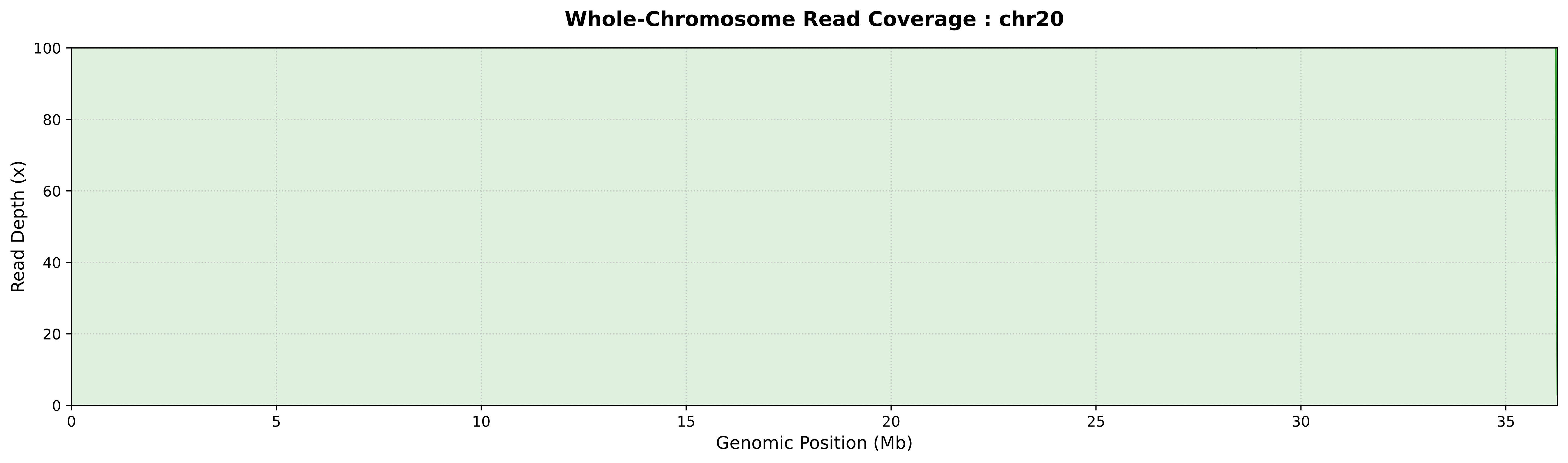

Whole-Chromosome Read Coverage : chr20
Read Depth (x)
0
20
40
60
80
100
Genomic Position (Mb)
0
5
10
15
20
25
30
35

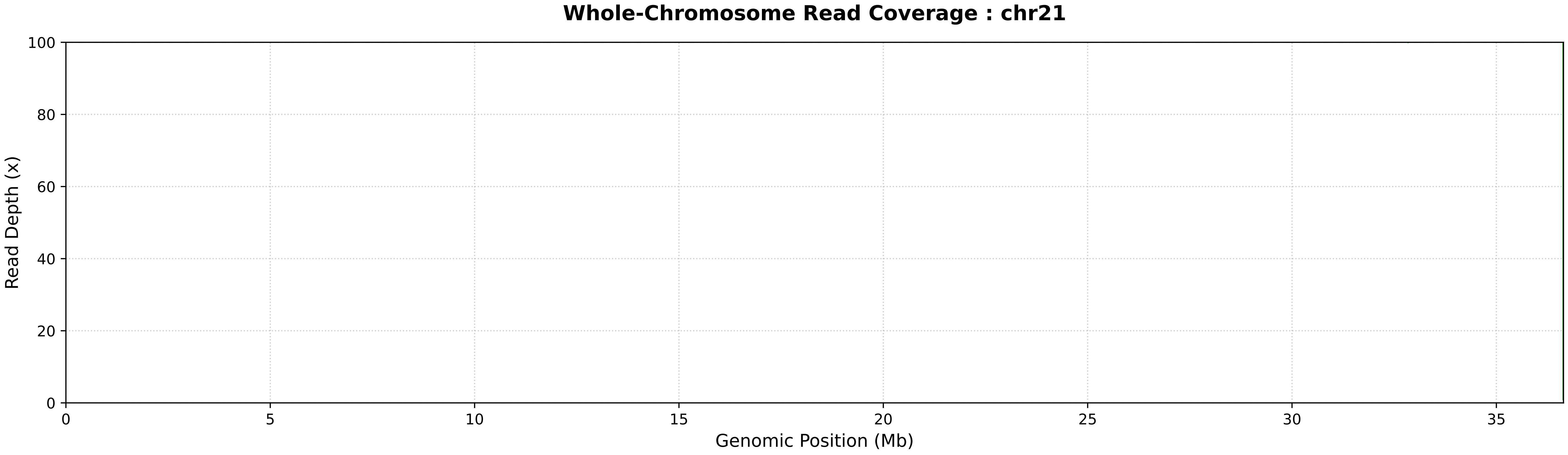
Whole-Chromosome Read Coverage : chr21
Read Depth (x)
0
20
40
60
80
100
0
5
10
15
20
25
30
35
Genomic Position (Mb)

Whole-Chromosome Read Coverage : chr22

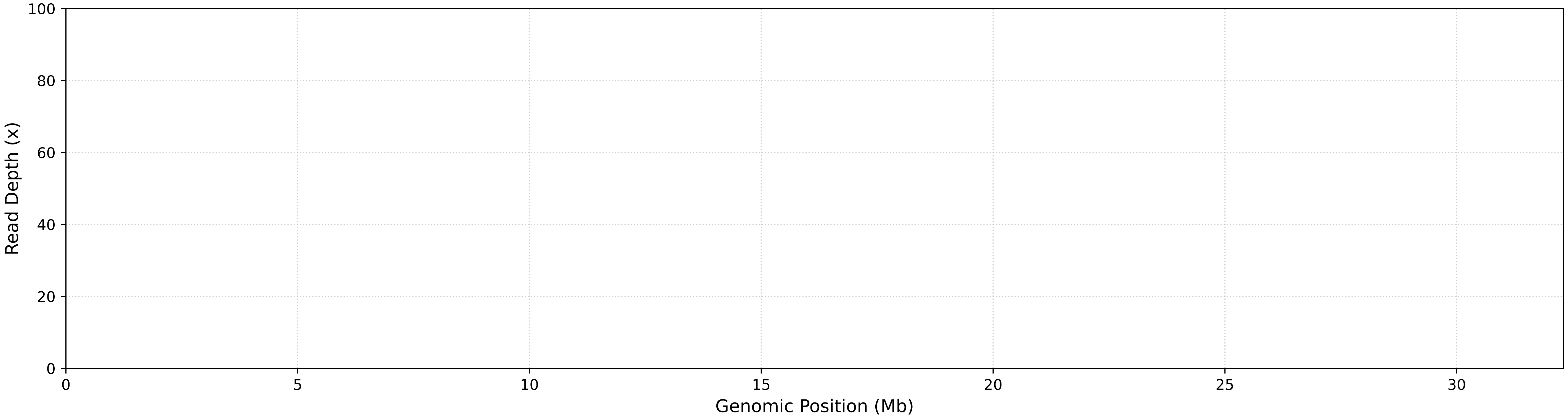

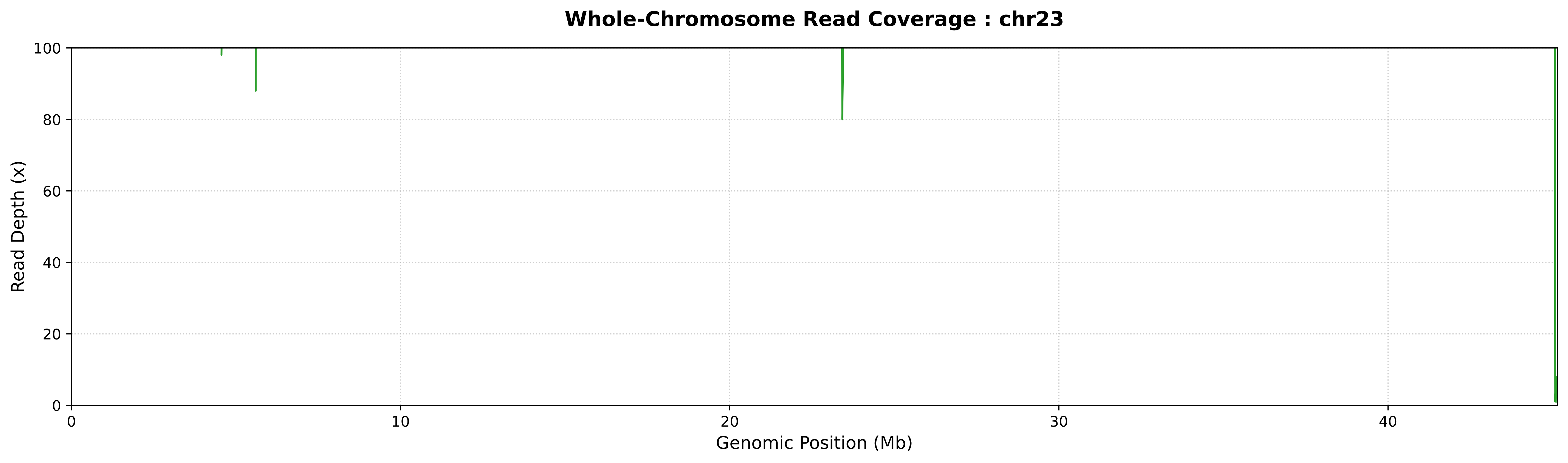
Whole-Chromosome Read Coverage : chr23
Read Depth (x)
0
20
40
60
80
100
0
10
20
30
40
Genomic Position (Mb)

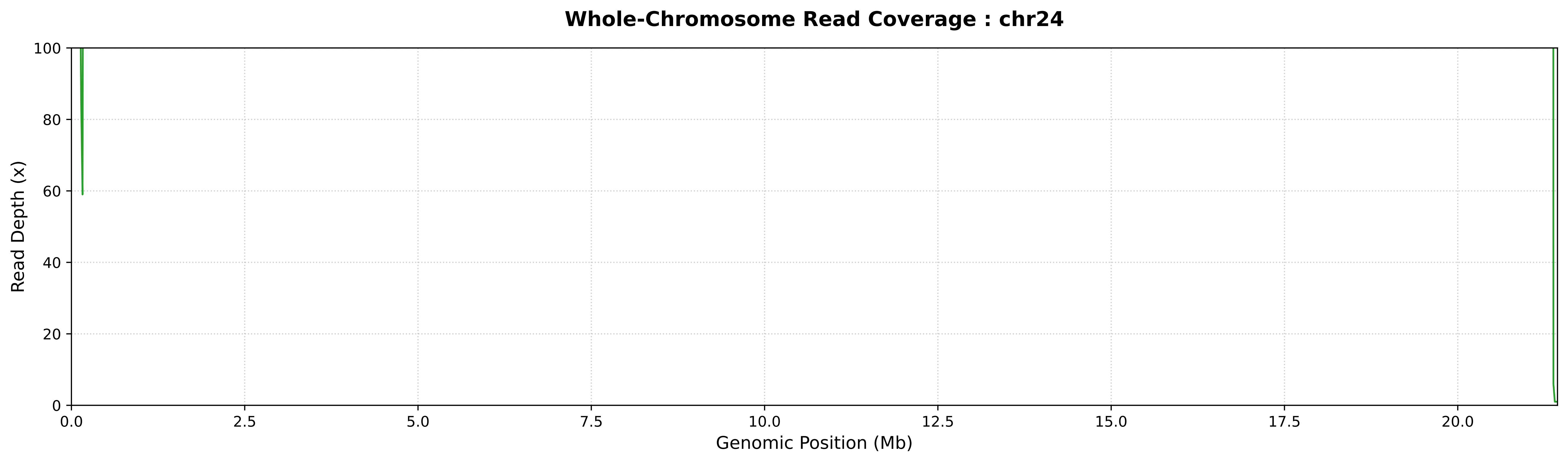
Whole-Chromosome Read Coverage : chr24
Read Depth (x)
Genomic Position (Mb)
0
20
40
60
80
100
0.0
2.5
5.0
7.5
10.0
12.5
15.0
17.5
20.0
